# Eurasian griffon vulture (*Gyps fulvus*) as a bone modifying agent and its implications for archaeology

**DOI:** 10.1038/s41598-023-44302-4

**Published:** 2023-10-10

**Authors:** Maite Arilla, Jordi Rosell, Antoni Margalida, Andreu Sansó, Ruth Blasco

**Affiliations:** 1 Institut català de Paleoecologia Humana i Evolució Social (IPHES-CERCA), Zona Educacional 4, Campus Sescelades URV (Edifici W3), 43007, Tarragona, Spain; 2 Departament d’Història i Història de l’Art, Universitat Rovira i Virgili (URV), Avinguda de Catalunya 35, 43002 Tarragona, Spain; 3 Instituto Pirenaico de Ecología (CSIC), Jaca, Huesca Spain; 4 Instituto de Investigación en Recursos Cinegéticos (IREC), CSIC-UCLM-JCCM, Ciudad Real, Spain; 5 Departament d’Economia Aplicada, Universitat de Les Illes Balears, Palma, Mallorca Spain; 6 Models for Information Processing and Fuzzy Information (MOTIBO) Research Group, Health Research Institute of the Balearic Islands, Idisba, 07120 Palma, Mallorca Spain

**Keywords:** Behavioural ecology, Palaeoecology, Animal behaviour

## Abstract

Neo-taphonomic studies have allowed us to detect bone damage patterns linked to carnivore preferences and behavioral traits as well as to improve our understanding of the origin of different alterations on vertebrate fossil faunas. However, taphonomically speaking vultures are among the least studied of all common, obligate scavengers. The research reported here contributes to characterise Eurasian griffon vulture (*Gyps fulvus*) behavior from a taphonomic perspective describing bone damage on 12 small-sized ungulate carcasses. The combination of observational data from photo/video-trap together with taphonomic analyses allowed us to manage factors like feeding behavior or time of consumption, as well as to accurately record bone modified items. Some bone-modifying effects are described here for the first time as vulture-made bone-damage distinctiveness. Still, some others may pose equifinality problems especially regarding small carnivores. This taphonomic conundrum leaves an interpretation problem particularly in archaeological sites in which those agents are present and consequently, an individualization dilemma about the taphonomic actors involved in bone modified assemblages.

## Introduction

Throughout the past few decades, bone modifying agents (e.g. mammalian carnivores, birds of prey) have been under consideration in most studies aiming to further explain their role in the formation of archaeological faunal assemblages. Hence, taphonomy has played a central task in our improved understanding of the natural and cultural processes involved in those sites and concurrently, neo-taphonomic and actualistic studies have been essential in expanding the criteria used to individualize those predators and find out diagnostic elements to differentiate them (e.g.^1–10^).

The role played by carnivores in the archaeological sites and their significance in the human behavior has led to most studies focusing on these predators (e.g.^5,11–18^), while only a few have focused their attention on different bone-modifying agents such as birds of prey^19–24^ and, of these, an even a smaller percentage have focused on vultures^25–28^. Those large carrion-eating birds are potential taphonomic agents in archaeological bone sets, since they have been documented in Eurasia during the Middle and Upper Pleistocene (e.g.^29–31^), not only in open air archaeological sites by consuming the remains accumulated by different taphonomic agency (e.g. hominins, carnivores) but also in cave/shelter archaeo-paleontological sites by disturbing or directly accumulating bone remains (e.g. bearded vultures *Gypaetus barbatus*).

Currently, the menaces facing bird of prey populations around the world are countless and the majority of species are under pressure to a lesser or greater extent^32,33^. In this sense, vultures as species with larger body sizes, low fecundity and high specialization are more likely to be endangered since their populations are more threatened with extinction if external forces affect their demographic parameters increasing their mortality rates^34,35^. This is the case of Old World vultures with 68% of species threatened^32,36^. This threatened status, coupled with their importance as providers of ecosystem services, has led to a substantial body of research in the field of behavioural ecology and conservation biology in recent years (e.g.^37–45^). For instance, forensic taphonomy has also enlarged this inquiry for the benefit of forensic anthropology^46,47^. The effects of vulture modification on human remains and by analogy on pigs (their internal anatomy, fat distribution, general lack of fur, and omnivorous diet are similarly analogous to those of humans) have been documented so as to examine timing and sequence of processes such as the rate of skeletonization, disarticulation, and dispersal of bone remains as well as crucial factors being the postmortem interval estimations (PMI) or time since death^26,48–50^. In this manner, vultures emerge in the role of taphonomic indicators that can help to better understand postmortem events and interval estimations at those scavengers’ modified scenes. Unfortunately, these studies are primarily focused on soft tissue decomposition and spatial dispersion without regard to bone-modifying effects.

Some preliminary examples of bone alteration by vultures were showed by Cáceres^25^ without delving too deep into the subject. The first researcher in describing the taphonomic effects of New World vulture scavenging was Reeves^26^, who documented relatively shallow scratches mostly on the crania and mandibles and linear surface scratches with no depth. Those types of marking were so shallow that intentional cleaning or weather exposure would distort or eliminate their presence. In this study, American black vultures (*Coragyps atratus*) and turkey vultures (*Cathartes aura*) waited approximately 24 h before starting to scavenge and between 3 and 27 h of feeding to completely skeletonize the pig carcasses. The main focus of the research was to determining accurate postmortem intervals (PMI) so as to better interpreting taphonomic events and animal scavenging on human remains. The results showed that the raptors would modify the scene, altering and dispersing bones as well as changing the rate of decomposition. In this case, domesticated pigs (*Sus scrofa*) were used since their internal anatomy, general lack of fur and omnivorous diet, are generally accepted as a human alternative. Taphonomically speaking, the results reported by Domínguez-Solera and Domínguez-Rodrigo^27^ were more accurate. The main goal of this study was to show the patterns of bone surface modification according to their frequency and anatomical distribution on a carcass scavenged by griffon vultures (*Gyps fulvus*). A complete female adult deer carcass was feed on to vultures in order to record their feeding behavior and the resulting bone damage. The results showed considerable bone dispersion while the rib cage, the skull and the limbs remained attached by the skin. As previously reported by Reeves^26^, the disarticulated process was a consequence of the competition to gain access to edible tissues. According to the authors, a diagnostic feature of these raptors is the location of most of the bone marks on the diaphysis of long bones. Almost 22% of the anatomical elements bore marks on their surface, mostly in the form of shallow irregular scores and striae. Several types of marks were described as punctures and circular or oval pits containing crushed bone inside which raised an obvious case of equifinality with mammalian carnivore tooth marks. Trying to enhance the body of research, Fetner and Sołtysiak^28^ exposed a bull head to a griffon vulture with the aim of recording the marks on the cranium and mandible, specifically their distribution, length and morphology. The marks were described as shallow single lines although sometimes clustered in groups forming V or L-shape which could eventually disappear due to different diagenetic agents. The mark breadth was less than 1 mm in all cases while 0.3 mm was the maximum depth. The results of all those works show particular marks, mostly in the form of irregular scores and sometimes forming specific shapes. However, all modifications are mostly described as shallow scratches without depth. The mere fact of cleaning the bones to proceed with their analysis or even their exposure to different diagenetic agents could make these types of marks disappear. Some of the features described in these works make such marks prone to being confused with trampling-related effects. Only in Domínguez-Solera and Domínguez-Rodrigo^27^ the problem of equifinality with carnivore tooth marks arises although focused on specific modifications such as pits bearing some crushing points. Unfortunately, those works remain insufficient to build the griffon vulture-made pattern either because of the scarce/narrow sample together with the lack of variables considered such as age categories or prey species variety. Here, we present the characterization of scavenger behavior from a neo-taphonomic perspective describing European griffon vulture taphonomic bone-signature on ungulate carcasses in the Catalan Pyrenees (Val d’Aran, Pallars Sobirà and Pallars Jussà).

## Materials and methods

### Study area and subject population

The study was carried out between summer 2022 and winter 2023 in the Pallars Jussà (Pre-Pyrenees, Catalonia). The region displays a diverse range of altitudes, spanning from approximately 500 to nearly 3000 m a.s.l. This variation in altitude contributes to significant climatic differences, resulting in contrasting landscapes and a rich array of vegetation. This region is sparsely populated by humans, who subsist there largely on a mixed economy, based mostly on animal husbandry and tourism. Mediterranean shrubland and mixed woodlands consisting of oak species (*Quercus ilex, Q. faginea*) and Scots pine (*Pinus sylvestris*) are the dominant vegetation types in the cultivated lands of the lower valleys, which are situated below 700 m a.s.l. Montane forest zones occur at the middle altitudes (700–1600 m a.s.l.), mainly dominated by red Scots pine, European beech (*Fagus sylvatica*) and a wide variety of scrublands, open grasslands and pasturelands. In the upper mountain areas (1600–1800 m a.s.l. upwards), mountain pines (*Pinus uncinata*) are abundant, and above the tree line (2300 m a.s.l.), a mosaic of different types of alpine pastures dominate^51^. The area is inhabited by the four obligate vulture species: Griffon vulture, Bearded vulture, Egyptian vulture (*Neophron percnopterus*) and Cinereous vulture (*Aegypius monachus*). Extensive and semi-extensively reared livestock (cattle, sheep and horses) provide most of the biomass for the scavenger guild^52^. Extensive livestock are kept outdoors from April to October, depending on the weather conditions, and some transhumant herds migrate to high summer pastures. Significant populations of herbivorous ungulates, such as red deer (*Cervus elaphus*), fallow deer (*Dama dama*), roe deer (*Capreolus capreolus*), and Pyrenean chamois (*Rupicapra pyrenaica*), inhabit the area. Of the obligate scavengers within a 30 km radius of the central study area, almost 800 pairs of griffon, 16 pairs of cinereous (*Aegypius monachus*), 21 pairs of bearded and 50 pairs of Egyptian vultures breed in the area^53^. Regarding facultative scavengers, the region exhibits a noteworthy population of avian (e.g., corvids and raptors such red kite *Milvus milvus* and black kite *Milvus migrans*) and mammal species. Additionally, shepherd dogs and hunting dogs that roam freely are frequently observed^37,53^.

The Griffon vulture is a cliff-nesting, socially monogamous colonial species that may nest in large colonies of over 150 pairs^54^. Both sexes incubate the eggs and feed the chicks with an equal division during the breeding season^55^. In the study area, the griffon vulture starts their breeding period in January–February coinciding with egg-laying. The incubation period is about 54–58 days and the chick (only one chick per pair is raised) remains at nest during 1 months (fledging take place in June–August). Both sexes have a similar role in parental roles incubating, and sharing chick rearing tasks proportionally as occurs on other vulture species^56^. Foraging areas are very extensive (hundreds of km) and individuals monitored with GPS transmitters in the study area can visit areas of Extremadura or Alps, as have been documented recently^45,57^.

### Vulture-derived sample

The remains of 12 small-sized ungulate carcasses (two *Equus caballus*, two *Bos taurus*, six *Sus scrofa*, one *Capreolus capreolus* and one *Ovis aries*) scavenged by vultures (henceforth abbreviated as OB[s], as each of them comes from a different observation) in the region of Pallars Jussà during summer and autumn months of 2022 and winter 2022/23 were analyzed. All carcasses used in our observations weight less than 100 kg and were deposited whole in the selected places. The carcasses were made available to the vultures by: (1) vulture scavenging of natural deaths; (2) road kills/required culling provided by the rangers and transported by ourselves to official bird-watching points. In the first case we were able to document vulture scavenging of carcasses on domestic animals because livestock owners notified rangers’ teams of those feeding bouts. Only in one observation (OB1) the carcass was unsupervised for a few hours at dawn so the possibility of some secondary scavengers exists. In the second case we were able to record exclusively vulture consumption since photo- and video trap systems were installed in the vicinity of carcasses. The ungulates were only exposed during daily time, from morning to sunset in order to prevent carnivore intervention at night. In a few cases, vultures did not access the remains the same day they were exposed, therefore, we covered the carcass during the night and uncover it the next morning. Thus, non-vulture scavenging was null and the whole process allowed us to record only vulture bone-damaging. Table 1 summarizes the main features of the neo-taphonomic study organized by individual observations.

**Table 1:** General characteristics of the experimental series organized by individual observations (OB).

OB	Season	Main scavenger	Secondary scavenger	Number of vultures	Exposure time*	Ungulate carcass	Age at death	Cause of death
OB1	Summer	*Griffon vulture*	*Vulpes vulpes*	40–45	2 days	*E. caballus*	Infantile	Unknown
OB2	Autumn	*Griffon vulture*	–	30–40	1 h	*Bos taurus*	Neonate	Stillborn
OB3	Autumn	*Griffon vulture*	–	30–35	2 h	*Sus scrofa*	Infantile	Road kill
OB4	Autumn	*Griffon vulture*	–	25–35	1 h	*Ovis aries*	Senile	Slaughtered
OB5	Winter	*Griffon vulture*	*Vulpes vulpes*	30–35	4 h	*Sus scrofa*	Infantile	Required culling
OB6	Autumn	*Griffon vulture*	*Aegypius monachus*	45–50	50 min	*C. capreolus*	Infantile	Road kill
OB7	Autumn	*Griffon vulture*	*Buteo buteo*	25–35	1 h	*Sus scrofa*	Infantile	Road kill
OB8	Autumn	*Griffon vulture*	*Aegypius monachus*	60–70	40 min	*Sus scrofa*	Juvenile	Road kill
OB9	Winter	*Griffon vulture*	*Vulpes vulpes*	30–35	2 h	*Sus scrofa*	Infantile	Required culling
OB10	Summer	*Griffon vulture*	–	40–45	5 h	*E. caballus*	Infantile	Unknown
OB11	Autumn	*Griffon vulture*	*Aegypius monachus*	55–65	45 min	*Sus scrofa*	Juvenile	Road kill
OB12	Autumn	*Griffon vulture*	–	25–35	1 h	*Bos taurus*	Neonate	Stillborn

*Total time from initial scavenging to final removal.

### Taphonomic analyses

All the skeletal remains were recovered and cleaned to expose bone surfaces using non-aggressive procedure, such as boiling in a mixture of water and non-enzymatic detergent. More resistant tissues were removed by using hair brushes and wooden/soft pointed tools that did not compromise the bone surface. Only ungulate carcasses weighting less than 100 kg were included in this case study; which includes different age groups, from neonate to senile.

Bone damage on the skeletal remains was analyzed using an Olympus SZ 11 stereoscopic (magnification up to 110), together with an ESEM (FEI QUANTA 600 Environmental Scanning Electron Microscope) when necessary. The bone surface modifications were documented combining the criteria defined by Maguire et al.^58^, Haynes^59^ and Blumenschine and Selvaggio^60^ for mammalian carnivores with those used by Armstrong and Avery^20^ and Lloveras et al.^23^ for birds of prey—on previous studies of Andrews^61^. In the case at hand, the vulture-induced bone damage was classified as follows:

1. Pits and punctures are marks caused when the tooth/beak (or teeth) of the taphonomic agent penetrates into (pitting) or through (puncturing) the cortical bone surface.
2. Scores are essentially elongated marks usually U-shaped and generated by dragging a tooth/beak along the surface of the bone.
3. Notches are angled marks usually without depth cut in the edge or top of the bone, usually recorded on flat bones.
4. Grooves described as a transversal narrow cut or depression in the surface of the bone that are divided here into: (1) general grooves as described right in the above line. (2) V-grooves which are channel-shaped notches usually located vertically/transversally on the ends of flat bones such as scapulae, ilium and ischium, mandibular angle (gonium) and, distal shafts of ribs, and especially in non-adult individuals belonging to large size (e.g. *E. caballus*). Their angular shape begins in the bone cortex and continues through the cancellous tissue with considerably depth and width angles. (3) U-grooves are described like the previous ones and located in the same skeletal elements, portions and individuals, and have been considered as notches in thinner cortical tissue. The difference between the two alterations (V–U) remains in their U-shape. This circular shape begins in the bone cortex and continues vertically/transversally through the trabeculae with considerably depth and relatively wide initial openings.
5. Crushing is produced by the breakdown of concentrated areas of cortical tissue in the form of short cracks and splits. We add to this study longitudinal crushing as extended longitudinal fractures located on the borders/edges of the thin cortical bone (e.g. spinous/transverse processes of vertebrae and rib shafts) with considerable length but narrow fracture width and conspicuous splits along the modification.
6. Crenulated edges often occur on flat bones in the form of notched and ragged fractures when the force applied overcomes the density and strength of the bone tissue.
7. Classic and general peeling usually occurs in bone elements susceptible to fracture by bending, such as ribs or vertebrae, although it can be documented on other skeletal elements, such as pelvis or scapulae. It can be identified by roughened surfaces where cortical layers have been stripped away and where cracks are present at the ends of the fractures with irregular shapes^62^. Incipient peeling includes fraying and bent ends on the cortical layers^63^.
8. Longitudinal cracks are fractures following the lines of bone collagen usually produced by the expansion of a perforation.
9. Fractures are complete breakages of cortical tissue. They appear as abrupt and clean interruptions of the bone structure, in such a way that the complementary ones can be recovered.
10. Hollow out is described here as an emptying of cancellous tissue similar to scooping out on epiphyseal sections but located in flat bones such as the scapulae (medial border), pelvis (ilium and ischium) and distal shafts of the ribs and mostly recorded in large-sized non-adult individuals. It is caused with the beak by dragging away soft bone tissue in those specific skeletal portions and leaving orifices in the form of conspicuous hollow punctures.

### Statistical analyses

We utilized a multinomial model to examine the bone taphonomic modifications, with a focus on predicting outcomes and emphasizing a comparative analysis among three distinct scavenger species namely *Gyps fulvus*, *Ursus arctos* and *Vulpes vulpes*. Although other classification techniques are available, such as regression trees and their derivatives or neural networks, to mention a few, we decided to use multinomial models since they are not black box models, as they allow an easy and clear interpretation of the results. Those models enable, in addition, quantification of changes in the probability of classification in the face of variations in the conditioning variables. That is, we are interested not only in classifying correctly, but also in learning what the determinants of classification are (see, for instance^64^). Moreover, the accuracy (i.e. out of sample percentage of correct classification) of the estimated multinomial logistic model outperforms that of the regression tree in our empirical case—the sample accuracy for the multinomial logistic model is 81.2% whereas that of the pruned regression tree is 79.6%. Using the tenfold cross validation method, where the sample is randomly divided into 10 parts and in 10 iterations 90% of the sample is used to estimate the model and the remaining 10% is predicted, gives an accuracy of 77.8% for the multinomial model versus 74.4% for the regression tree. Using the Leave-One-Out cross-validation method, the multinomial model gives an accuracy of 77.7% whereas that of the pruned regression trees is 74.1%.

Suppose we have a response variable Y that can take on one of K possible outcomes, with K > 2. In our case, we observed the three categories of scavengers, so that K = 3. Also, we observed a *p*-dimensional vector, X, of characteristics of a damaged bone (number of pits, scores, fractures, type of bone, damaged area, etc.). A multinomial model seeks to determine the probability of Y taking a specific value, given X. This is mathematically denoted as P(Y = k|X). In the case of multinomial logistic regression, one of the most common approaches, one models the log-odds as a linear function of X. In this model, the conditional probability of Y given X is:

$$P\left( {Y_{i} = k|X_{i} } \right) = \frac{{exp\left( {X_{i} \beta_{k} } \right)}}{{\mathop \sum \nolimits_{j = 1}^{K} exp\left( {X_{i} \beta_{j} } \right)}}$$

for *k* ∈ {1,…,K}, where $\beta_{k}$ is a vector of parameters conformable with the dimension of *X*_*i*_, *i* ∈ {1,…,*n*}, and *n* is the number of observations. Given that if we make $\beta_{k}^{*} = \beta_{k} + \gamma$ for all *k* we get the exactly the same probabilities, it is usual to normalize the model taking $\beta_{1} = 0$, the *Gyps fulvus* in our setting. Thus, we may write

$$P\left( {Y_{i} = 1|X_{i} } \right) = \frac{1}{{1 + \mathop \sum \nolimits_{j = 2}^{3} exp\left( {X_{i} \beta_{j} } \right)}},$$

$$P\left( {Y_{i} = k|X_{i} } \right) = \frac{{exp\left( {X_{i} \beta_{k} } \right)}}{{1 + \mathop \sum \nolimits_{j = 2}^{3} exp\left( {X_{i} \beta_{j} } \right)}}$$

for *k* ∈ {2,3}. Then, the log of the odds is linear

$$ln\frac{{P\left( {Y_{i} = k|X_{i} } \right)}}{{P\left( {Y_{i} = 1|X_{i} } \right)}} = X_{i} \beta_{k} = \beta_{k,0} + \beta_{k,1} x_{1i} + \cdots + \beta_{k,p} x_{pi}$$

for *k* ∈ {2,3}, $x_{ji}$ is the value of variable/characteristic *j* (*j* ∈ {1,…,*p*}) in observation *i* (*i* ∈ {1,…,*n*}), $\beta_{k,j}$ is its associated coefficient of this variable in category *k*, and $\beta_{k,0}$ is a constant term. For quantitative variables, the value of $\beta_{k,j}$ should be interpreted as the effect of an increase of one unit in the log-odds, whereas for qualitative variables, which are introduced in the model using dummy-variables, they should be interpreted as the effect on the log-odds from moving from the base category of the qualitative variable to the category indicated by the dummy variable. The base category is *no-damage* for all of the qualitative variables used in our model except *Age* and *Skeletal group*. If we want to compare the log-odds of categories 2 and 3,

$$ln\frac{{P\left( {Y_{i} = 3|X_{i} } \right)}}{{P\left( {Y_{i} = 2|X_{i} } \right)}} = X_{i} \left( {\beta_{3} - \beta_{2} } \right) = \beta_{3,0} - \beta_{2,0} + \left( {\beta_{3,1} - \beta_{2,1} } \right)x_{1i} + \cdots + \left( {\beta_{3,p} - \beta_{2,p} } \right)x_{pi}$$

and we have to look at the difference among the coefficients. The most common way to estimate the parameters of a multinomial model is through the method of maximum likelihood. All the computations and data analysis were performed in R language version 4.3.0 (R Core Team 2023; www.R-project.org/) and using package *nnet*^65^ and *ggplot2*^66^.

## Results

The total osteological assemblage from all 12 small-sized ungulate carcasses scavenged by griffon vultures displays a diversity of scavenged-related bone damage, including tooth pits/punctures and scores, notches, grooves, crushing, crenulation, fracturing, peeling, longitudinal cracking and hollow out (Table 2). Of the total number of recovered skeletal specimens (n = 1354), 270 (19.9%) exhibit at least one occurrence of bone alteration, with most of the altered specimens deriving from bones of the cranial (n = 20; 55.6%) and girdle (n = 23; 51.1%) regions followed by the axial skeleton (n = 221; 43.8%). Only six limb specimens (3.4%) bear griffon vulture- induced damage (Table 3).

**Table 2:** Recovered bones vs damaged bones by skeletal elements split by different bone damage type.

Skeletal element	Rcv Bn (n)	Dg Bn (%)	Pt, Pct, Sc n (%)	Ntc	Grv	TFrac	Crs	LCracks	Hllw	Pl	CrEdg
Skull	12	66.7	8 (66.7)	1 (8.3)	–	1 (8.3)	–	–	1 (8.3)	–	–
Hemi-mandible	24	50.0	4 (16.7)	–	7 (29.2)	1 (4.2)	2 (8.3)	–	–	–	1 (4.2)
Hyoid bone	4	50.0	–	–	–	2 (50)	–	–	–	–	–
Cervical vertebra	49	40.8	4 (8.2)	2 (4.1)	1 (2)	7 (14.3)	13 (26.5)	–	–	–	1 (2)
Thoracic vertebra	129	42.6	10 (7.8)	2 (1.6)	7 (5.4)	21 (16.3)	23 (12.4)	4 (3.1)	6 (4.7)	1 (0.8)	2 (1.6)
Lumbar vertebra	70	82.9	5 (7.1)	–	7 (10)	29 (41.4)	28 (25.7)	1 (1.4)	2 (2.9)	15 (21.4)	3 (4.3)
Caudal vertebra	13	7.7	–	–	–	–	1 (7.7)	–	–	–	–
Sacral vertebra	21	33.3	2 (9.5)	–	–	4 (19)	1 (4.8)	–	–	–	1 (4.8)
Sternum	7	14.3	1 (14.3)	–	–	–	1 (14.3)	–	–	–	–
Rib	201	38.3	16 (8)	4 (2)	7 (3.5)	20 (10)	22 (4)	7 (3.5)	8 (4)	8 (4)	–
Scapula	23	52.2	3 (13)	–	2 (8.7)	–	1 (4.3)	2 (8.7)	2 (8.7)	–	5 (21.7)
Humerus	24	4.2	1 (4.2)	–	–	–	–	–	–	–	–
Hemi-pelvis	22	50.0	4 (18.2)	–	6 (27.3)	1 (4.5)	6 (18.2)	1 (4.5)	1 (4.5)	–	–
Femur	24	16.7	4 (16.7)	–	–	–	–	–	–	–	–
Tibia	24	4.2	1 (4.2)	–	–	–	–	–	–	–	–
Total	647	41.7	63 (9.7)	9 (1.4)	37 (5.7)	86 (13.3)	98 (15.1)	15 (2.3)	20 (3.1)	24 (3.7)	13 (2)

Only the skeletal categories with specimens bearing inflicted-damage are registered in the table.

Note that some specimens show co-occurrence of damage and therefore, the total number (summing up all types) can be higher in some categories.

Rcv Bn, Recovered bones; Dmg Bn, Damaged bones; Pt, Pct, Sc, Pits, Punctures, Scores; Ntc, Notches; Grv, Grooves; TFrac, Transversal Fractures; Crs, Crushing; LCracks, Longitudinal cracks; Hollow out, Hllw; Pl, Peeling; CrEdg, Crenulated edges.

**Table 3:** Number of recovered ungulate skeletal elements and damaged bones in the aggregated vulture-modified sample by OB and anatomical regions.

Carcass	OB	Recovered bones (n)							Damaged bones (%)						
		CR	AX	GD	LB	BSPD	ACRPD	PT/SSM	CR	AX	GD	LB	BSPD	ACRPD	PT/SSM
*E. caballus*	OB 1	3	28	3	14	24	12	16	3 (100)	20 (71.4)	1 (33.3)	3 (21.4)	–	–	–
*Bos taurus*	OB 2	3	64	4	14	24	24	2	3 (100)	20 (31.2)	–	–	–	–	–
*Sus scrofa*	OB 3	3	46	4	14	24	24	2	2 (66.6)	32 (69.6)	4 (100)	–	–	–	–
*Ovis aries*	OB 4	3	65	4	16	24	24	2	1 (33.3)	12 (18.5)	1 (25)	1 (6.2)	–	–	–
*Sus scrofa*	OB 5	3	5	3	11	18	18	2	1 (33.3)	5 (100)	3 (199)	–	–	–	–
*C. capreolus*	OB 6	3	31	4	16	24	24	2	3 (100)	26 (83.9)	3 (75)	–	–	–	–
*Sus scrofa*	OB 7	3	22	4	16	24	24	2	–	6 (27.3)	1 (25)	–	–	–	–
*Sus scrofa*	OB 8	3	50	4	16	24	24	2	–	17 (34)	2 (50)	–	–	–	–
*Sus scrofa*	OB 9	3	23	4	14	24	24	2	3 (100)	22 (95.6)	3 (75)	–	–	–	–
*E. caballus*	OB 10	3	58	4	14	24	12	16	2 (66.6)	46 (79.3)	4 (100)	2 (14.3)	–	–	–
*Sus scrofa*	OB 11	3	43	3	16	24	24	2	–	14 (32.6)	1 (33.3)	–	–	–	–
*Bos taurus*	OB 12	3	69	4	16	24	24	2	2 (66.6)	1 (1.4)	–	–	–	–	–
Total		36	504	45	177	282	258	52	20 (55.6)	221 (43.8)	23 (51.1)	6 (3.4)	–	–	–

*CR* Cranial; *AX* Axial; *GD* Girdle; *LB* Limbs; *BSPD* Basipodials; *ACRPD* Acropodials; *PT/SSM* Patella/Sesamoideum.

**Figure 1:**
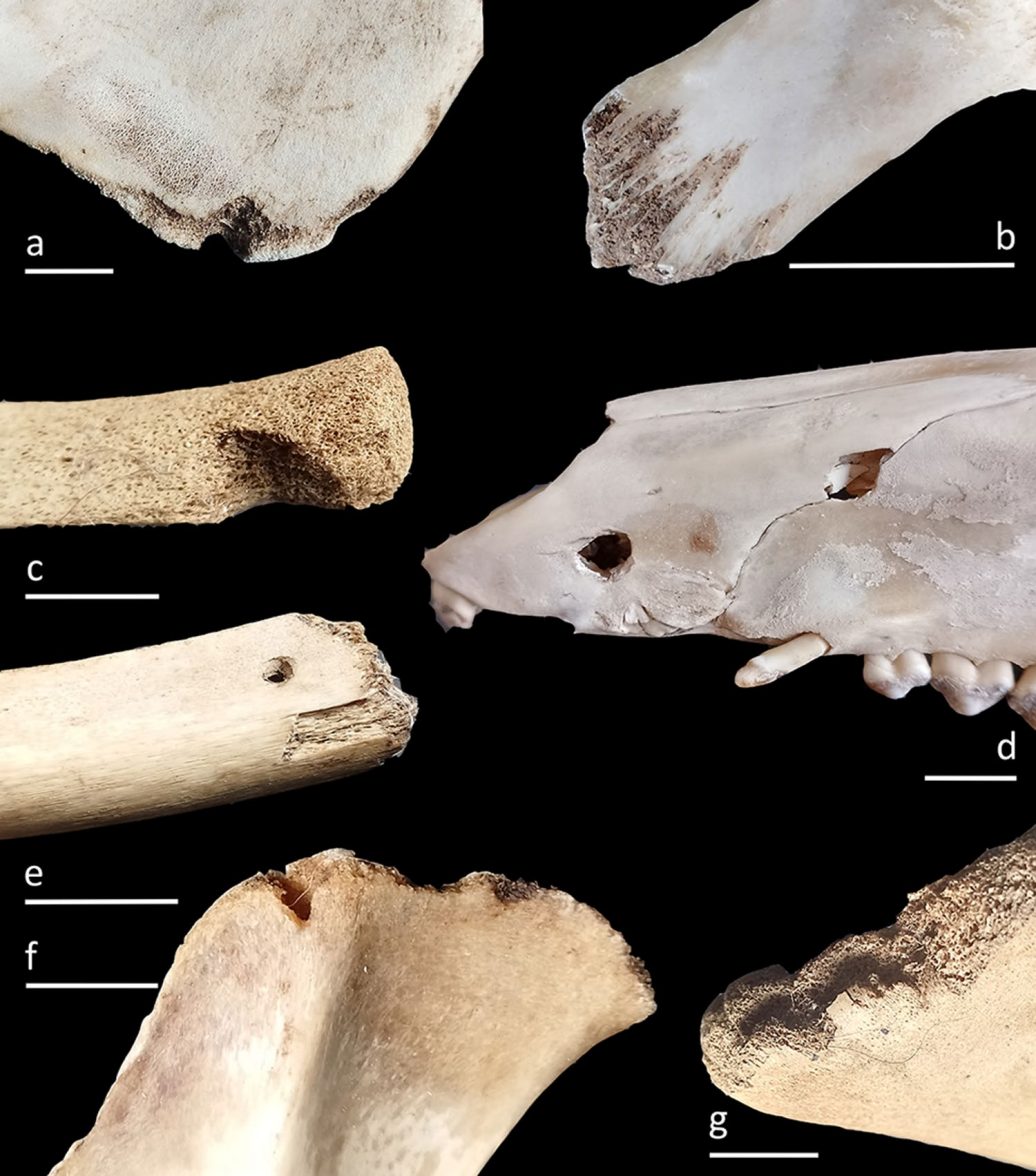
(**a**) V-groove on the mandibular angle of a calf (OB2); (**b**) Classic peeling on the transverse process of a lumbar vertebra (OB3); (**c**) U-groove on the distal shaft of a rib (OB10); (**d**) Punctures on the maxilla of a wild boar (OB14); (**e**) Co-ocurrence of a pit and classic peeling (incipient) on the distal shaft of a rib (OB10) (**f**) V-groove on the ilium of a pelvis (OB6); (**g**) Hollow out on the medial border of a scapula (OB10). Scale: 1 cm.

**Figure 2:**
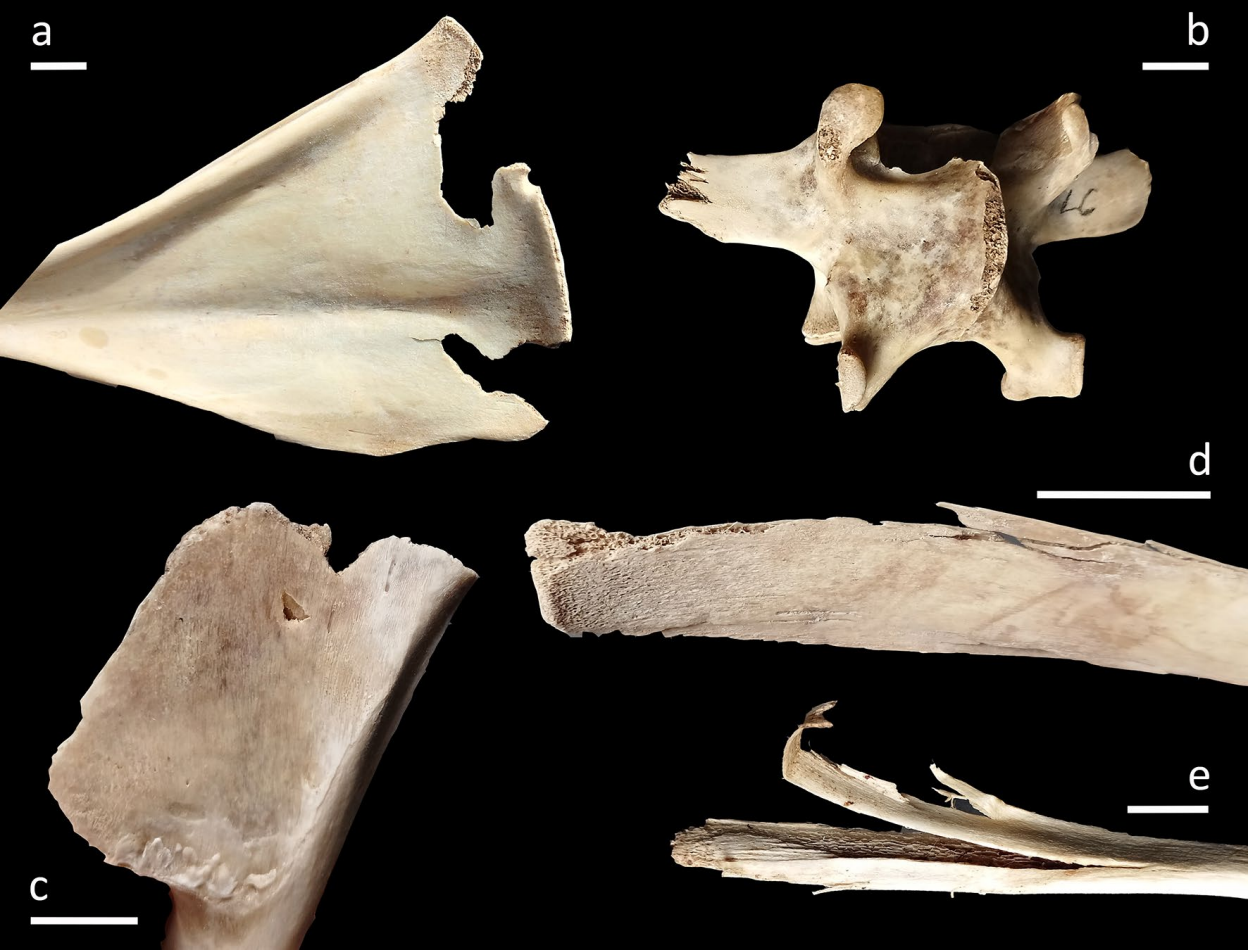
(**a**) Crenulated edge on the medial border of a scapula (OB14); (**b**) Fractures on the spinous and transverse processes and puncture on the superior articular facet of a vertebra (OB14); (**c**) V-groove associated to an angular pit on the ilium region of a pelvis (OB13); (**d**) Longitudinal crushing on a rib of a roe deer (OB6); (**e**) Fraying on a rib of a calf (OB2). Scale: 1 cm.

**Figure 3:**
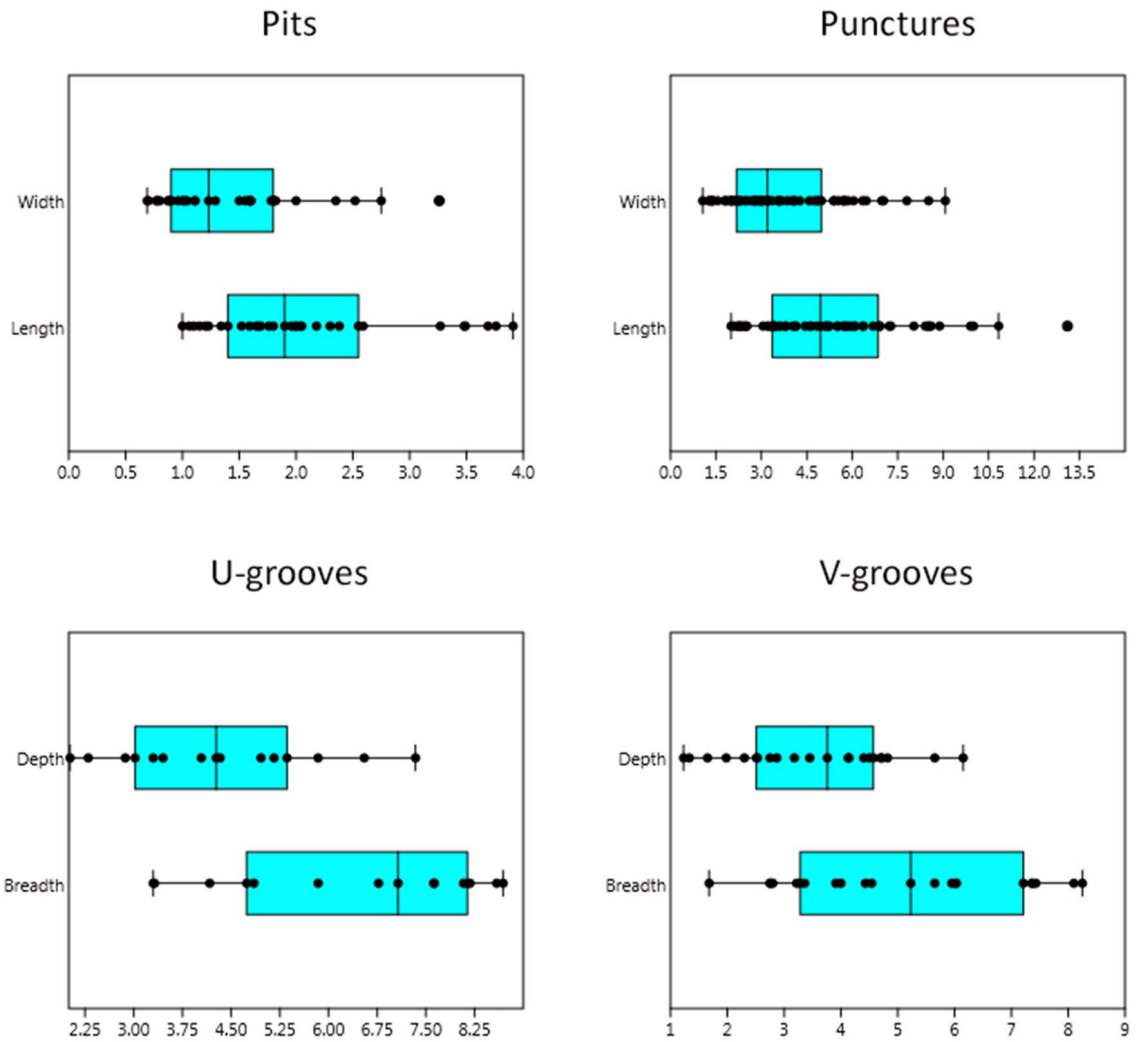
Box plots show the measurements of pits/punctures (width and length), and the dimensions of U- and V-grooves (depth and breadth) occurring on thin cortical bone.

**Figure 4:**
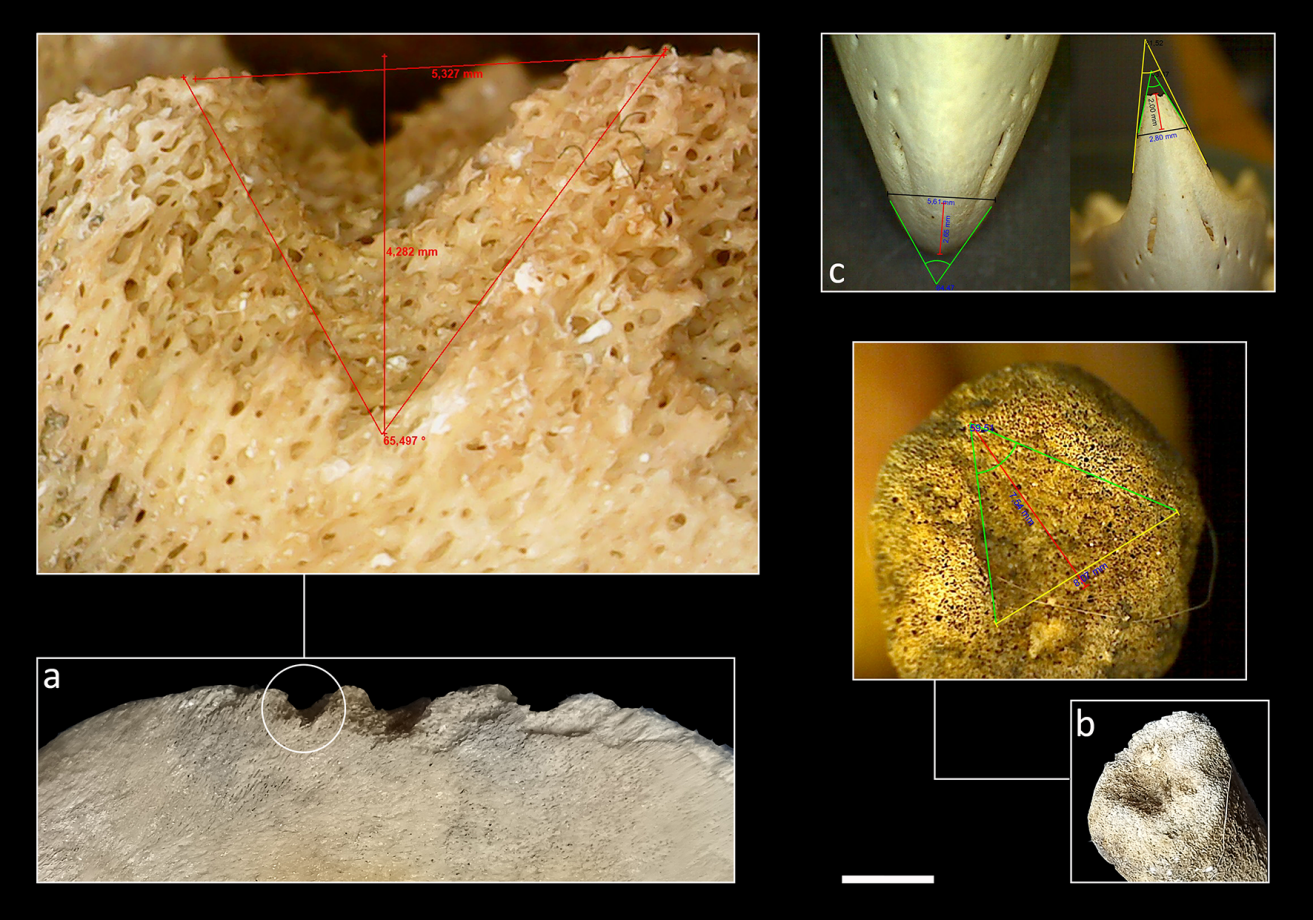
Some examples of v-grooves: (**a**) Mandibular angle of a foal (OB1); (**b**) Hollow out with angular shape on the costochondrale articulation of a rib (OB1); (**c**) Measurement of different angles depending on the depth of the vultures beak. Scale: 1 cm; magnification: (**a**) × 3.55; (**b**) × 3.22.

Here we present the overview of bone surface modification incidences and anatomical locations. Note the co-occurrence of more than one alteration on the same specimen:

1. Pits/punctures and scores are fairly common in the vulture-created sample with 63 affected-skeletal specimens (9.7%) and the most significant values recorded on the skull (66.7% related to recovered specimens of the same anatomical regions). These are the only alterations documented on long limb bone specimens with the femur being the most affected skeletal element (16.7%) followed by humerus and tibia (4.2% both). With regard to pits/puncture beak marks we emphasize on the distinction between both marks as a way to individualize/differentiate different taphonomic agents in the case of equifinality problems. Punctures are recorded on 39 bone elements (6%) whereas pits are documented on seventeen specimens (2.6%). Among the observed beak marks, a percentage of 58.3% cranial specimens are punctured while pits are recorded with values of 16.7% on the same anatomical region. The maxilla (n = 3), the hard palate (n = 3) and the sphenoid (n = 3) exhibit the highest number of punctures among these anatomical regions (Fig. 1d). Hemi-mandible (n = 2), sternum (n = 1) and femur (n = 3) bear only puncture marks on its ramus, body and diaphysis/epiphyses respectively. Six hemi-pelvises are punctured (n = 3) and pitted (n = 3) as well as nine (4.5%) and four (2%) ribs are punctured and pitted respectively. In this case, two pits and three punctures are documented on the costochondral joint whereas the rest (n = 5 punctures and n = 2 pits) occurs predominantly on the distal shafts of the ribs. Twelve vertebrae are punctured (seven thoracic, three lumbar and two cervical) and seven are pitted (four thoracic, two lumbar and one cervical) with most puncturing and pitting damage on the spinous processes and much more rarely on transverse processes. Only one sacral vertebra shows this type of modification (1 pit) on its spinous process and a sternum (1 puncture) on its body. Regarding exclusively pit marks we recorded a total of 0.8% of angular shaped pits (n = 5) located on three ribs, one lumbar vertebra and one hemi-pelvis (Fig. 2c). The size of those beak marks ranges from 1 × 0.7 mm to 5.3 × 5.26 mm with an average of 2.1 × 1.5 mm in the case of pits and 2 × 1.1 mm to 13.1 × 9.1 mm with the average of 5.2 × 3.7 mm regarding puncture marks (Fig. 3). All pits/puncture beak marks were located on thin cortical tissue with the exception of one puncture recorded on the body of a sternum and three punctures documented on the proximal diaphysis (n = 1), distal diaphysis (n = 1) and distal epiphyses (n = 1) of one femur.
2. Scores are detected on 2.3% (n = 15) of the sample with the cranial region showing the highest incidence at 8.3%. These scores are located on both the maxillary body (skull) and ramus (mandible). The appendicular region is also affected with a 4.2% of scoring specifically on one femur, one tibia and one humerus. Two cervical vertebrae and one sacral vertebra bear scores mainly on its transverse process, and the superior border of one scapula is also scored. Eventually, four ribs bear this alteration on the mid-shaft (n = 2), distal shaft (n = 1) and costochondral articulation (n = 1). Most of the skeletal elements exhibit shallow scores with random directions especially on appendicular specimens. Those marks are manly superficial with 2.7 mm and 0.2 mm in the maximum and minimum breadth size respectively and an average of 0.8 mm.
3. Notches are hardly represented with 1.4% (n = 9) of the sample especially on the maxilla of one skull (8.3%). In relation to the axial skeleton, four ribs display notches on the distal shaft (n = 3) and costochondrale articulation (n = 1); two thoracic vertebrae (4.1%) bear notches on the transverse process and the superior articular facet and two lumbar vertebrae (1.6%) show this type-mark on the border of the spinous process.
4. Grooves are moderately represented in the assemblage with a total of 37 (5.7%) modified items. V-groove beak marks are recorded on 21 (3.2%) skeletal elements and are included in the previous general percentage. This subtype is mostly documented on the angle of hemi-mandibles (n = 6) and the ilium of hemi-pelvises (n = 3) (Figs. 1a, f, 2c and 4a). Five ribs contain this alteration basically on the distal shaft, and the transverse (n = 2) and the spinous (n = 3) processes of the vertebrae are also v-grooved. One scapula shows v-grooves on its medial border. The hooked tip’s sharp drop of the vulture’s beak due to its angular shape and the mobility of the neck would be the cause of these modifications (Fig. 4c). The u-grooves are observed on 14 items (2.2%) that are also included in the general groove percentage. Innominate bones bear the highest percentage (13.6%) of this inflicted mark mainly on its ilium and the angle, and the condyle of two hemi-mandibles are also u-grooved (8.3%). Two scapulae (8.7%) exhibit u-grooves on the medial border and one single rib shows this mark on its distal shaft (Fig. 1c). Regarding the spine, the damage is located on the spinous process, laminae and superior articular process of three thoracic vertebrae and one transverse process, one spinous process and one body of three lumbar vertebrae. This particular shape has to be given by the slight upwards beak’s curvature. General grooves are registered on 6 specimens (0.9%). Concerning the axial skeleton, three lumbar vertebrae (4.3%) are grooved on the transverse (n = 2) and spinous (n = 1) processes and the spinous process of one thoracic vertebra (0.8%) has also this beak mark. One hemi-mandible bears 4 grooves on the angle and one rib is grooved on its costochondrale joint. The dimension of v-groove marks ranges from 1.7 mm × 1.2 mm to 8.3 mm × 6.2 mm with an average range of 5.1 mm × 3.5 mm regarding to v-grooves and from 3.3 mm × 2 mm to 8.7 mm × 7.3 mm with the average of 6.5 mm × 4.3 mm in relation to u-grooves (Fig. 3). The angles measured in relation to v-grooves range from 45° to 83°. Depths vary from 1.65 mm to 11.18 mm with the average of 4.4 mm regarding to v-grooves and from 2.02 mm to 7.34 mm with an average of 4.5 mm in relation to u-grooves (see Fig. 4a).
5. Crushing is the most common type of bone alteration in the griffon vulture-modified sample occurring predominantly on the axial skeleton (n = 89) with the exceptions of hemi-pelvis (n = 6; 27.3%), hemi-mandible (n = 2; 8.3%) and scapula (n = 1; 4.3%). Common crushing (9.7%) is located exclusively on cervical (n = 13) and caudal (n = 1) vertebrae, hemi-mandible (n = 2) and sternum (n = 1), while longitudinal crushing (5.7%) is solely inflicted on one scapula and one sacral vertebra. Both subtypes, common crushing and longitudinal crushing, co-occur on thoracic and lumbar vertebrae (n = 16 and n = 7; n = 18 and n = 12 respectively), ribs (n = 8 and n = 14 respectively) and hemi-pelvises (n = 4 and n = 2 respectively). Transverse and spinous processes are highly affected with lumbar vertebrae on their transverse processes being the most affected item followed by cervical and thoracic vertebrae. The distal and mid-shafts of the ribs are mainly longitudinally crushed (Fig. 2d). Two hemi-mandibles are commonly crushed on its angle and six hemi-pelvises show this modification particularly on the ilium.
6. Fractures induced by griffon vultures in the form of crenulated edges are recorded on 13 (2%) specimens. These modifications are observed on the girdles, with the scapulae being the only altered skeletal element (n = 5; 21.7%) which is affected on its medial border (Fig. 2a), spine and supraspinous and infraspinous fossa. One hemi-mandible (4.2%) is crenulated on its posterior border of ramus regarding to the cranial skeleton. Vertebrae are the only bone specimens crenulated in relation to the axial skeleton, including the sacral and lumbar vertebrae (4.8% and 4.3% respectively), as well as the cervical and thoracic (2% and 1.6% respectively) vertebrae.
7. Although being registered mainly in primate produced faunas (e.g.^63^) and bear-modified samples^5,10^, we were able to document a relatively modest yet unexpected percentage (3.7%) of peeling damage in the griffon vulture-modified assemblage. Classic peeling is the most common type (n = 10) primarily affecting lumbar vertebrae (n = 5) with substantial damage on the transverse processes (n = 5) (Fig. 1b) and, to a lesser extent, on the spinous processes (n = 3). Additionally, four ribs exhibit peeling damage on their distal shafts (Fig. 1e). General peeling is documented on four lumbar vertebrae specifically on transverse (n = 4) and spinous (n = 2) processes and two ribs bear this type on damage on its head (n = 2) and distal shaft (n = 1). Seven lumbar vertebrae are incipiently peeled (bent end) on the transverse process and two ribs show fraying on the distal and mid-shaft (Fig. 2e).
8. Fifteen specimens are longitudinally cracked (2.3%) clustering on axial skeleton and girdles. Regarding the former, thoracic (n = 4) and lumbar (n = 1) vertebrae display this alteration exclusively on the spinous process and seven ribs (3.5%) are cracked on its distal (n = 4) and mid-shaft (n = 3). Concerning the latter, two scapulae (8.7%) show longitudinal cracks on the supraspinous fossa and, the ilium of one (4.5%) hemi-pelvis is also cracked.
9. Transverse fractures are recorded mainly on the hyoid bone (n = 2; 50%) and spinous and transverse processes of lumbar vertebrae (n = 29; 41.4%) (Fig. 2b). Thoracic and cervical vertebrae are also fractured on its transverse and spinous processes (16.3% and 14.3% respectively) together with sacral vertebrae (19%). Ten rib specimens are fractured mostly on the distal shaft (n = 12), mid-shaft (n = 3), proximal shaft (n = 3), head (n = 4), neck (n = 1) and tubercle (n = 1). Fractured portions of vertebrae and ribs, especially transverse/spinous processes and distal/mid-shafts are also related to the intra-specific vulture competition in foraging soft tissues quickly. The rest of the fractured specimens include one skull with a split on its styloid process, one breakage on the ilium of a hemi-pelvis and one fractured coronoid process of a hemi-mandible.
10. Hollow out is registered on 3.1% of the sample (n = 20). This alteration is mainly observed on the axial skeleton, particularly on the transverse and spinous processes of thoracic (n = 6) and lumbar (n = 2) vertebrae, as well as on the distal shafts of the ribs (n = 8). Additionally, one head and one costochondrale articulation are also hollowed out (Fig. 4b). Regarding girdle elements, scapulae (n = 2) show the highest percentages of this modification recorded on the medial border, and one hemi-pelvis is hollowed out on its ilium (Fig. 1g). One skull (8.3%) bears also this vulture induced-damage on both mastoid processes.

When split by age categories, the highest percentages of bone induced-damage belong to infantile individuals (n = 195; 27.8%) followed by juvenile (n = 34; 14.3%), senile (n = 15; 10.9%) and neonate ungulates (n = 26; 9.4%). Although neonates have the minimum general percentage, its cranial region shows the highest induced-damage with 83.3% of alterations specifically grooves and scores on the mandibular angle and punctures on the eye socket. We recorded 73.7% and 73.1% of vulture inflicted-damage on the axial and girdle areas (n = 157 and n = 19 respectively) of infantile individuals. In relation to the axial region, 105 vertebrae, 51 ribs and one sternum are damaged, as well as ten scapulae and nine hemi-pelvises that are also modified. The cranial area shows a significant degree of bone damage (66.7%) regarding this same age category, and limbs bear 5.1% of scoring mainly. The alterations with the highest percentages of beak marks inflicted on skeletal specimens of this same age category are crushing (10.1%), fractures (9.8%) and pits/punctures/scores (6.6%). Bone damage reaches 42.9% (n = 3) on the girdles and 33.3% (n = 31) on the axial portions of juvenile ungulates whereas no cranial or limb bones are modified. All anatomical regions of senile individuals present moderate vulture activity with the cranial region displaying 33.3% (n = 1) of bone modifications followed by girdles (n = 1; 25%), axial skeleton (n = 12; 18.5%) and limbs (n = 1; 6.3%). Respecting ungulate carcass species, a notable more intensive percentage of modifications is observed on *E. caballus* (35.1%) and *C. capreolus* (31.4%) followed by *S. scrofa* (19.2%), whereas *O. aries* (10.9%) and *B. taurus* (9.4%) seem to have slightly less intensive damage. There seems to be a deeper scavenging on summer (35.1%) and winter (24%) than on autumn (15.7%), although when split by skeletal parts we found the axial region (n = 27; 96.4%) to be the most exploited area in winter followed by girdles (n = 6; 85.7%) in the same year period. We found a general decrease in scavenging activity during autumn for all skeletal regions, including cranial (45.8%), girdle (38.7%), axial (32.8%) and limb bones (0.8%).

The multinomial logistic regression model was applied to the complete dataset consisting of 1782 observations which include data from neotaphonomic studies on brown bears^5^ and red foxes^6^. Table 4 presents the maximum likelihood estimates derived from this analysis, taking the griffon vulture as the reference category (i.e. $Y_{i} = 1$), and 9 quantitative conditioning variables (number of pits, scores, fracture, etc.) as well as 43 qualitative dichotomous ones (categories of age, skeletal group, and damaged areas). It also shows the standard errors and the associated *p*-value of each estimate.

**Table 4:** Estimated multinomial logistic regression.

	*Ursus arctos*		*p*-value	*Vulpes vulpes*		*p*-value
	Coeff	s.e		Coeff	s.e	
Intercept	22.818	0.439	0.000	18.603	0.632	0.000
N.Pits	− 0.032	0.119	0.790	0.299	0.109	0.006
N.Scores	− 0.872	0.316	0.006	− 0.816	0.317	0.010
N.Fractures	0.630	0.424	0.137	− 1.071	0.587	0.068
N.Crushing	1.181	0.357	0.001	0.314	0.545	0.565
N.Long.Crushing	− 24.887	1.8E− 10	0.000	− 18.982	1.2E− 07	0.000
N.Gral.Peeling	0.185	1.287	0.885	− 21.821	0.605	0.000
N.Bent.End	− 16.961	0.359	0.000	− 16.874	0.363	0.000
N.Cren.Edge	0.605	1.274	0.635	− 0.059	1.291	0.964
N.Furrowing	24.153	0.154	0.000	23.674	0.154	0.000
Classic.peeling	1.017	0.512	0.047	0.502	0.557	0.368
Fraying	20.421	0.120	0.000	20.389	0.120	0.000
Long.Cracks	− 0.425	0.488	0.383	0.014	0.490	0.977
Age.inf	− 24.518	0.239	0.000	− 23.108	0.267	0.000
Age.juv	− 22.324	0.266	0.000	− 19.416	0.272	0.000
Age.neo	− 57.596	7.2E−09	0.000	− 51.341	2.6E−07	0.000
Age.sen	− 22.127	0.385	0.000	− 39.836	4.3E−07	0.000
Skeletal.group.Cervical vertebra	0.948	0.634	0.135	2.426	0.824	0.003
Skeletal.group.Cranial skeleton	− 1.314	0.732	0.073	0.700	0.937	0.455
Skeletal.group.Girdles	− 1.222	0.679	0.072	− 0.065	0.904	0.943
Skeletal.group.Lumbar vertebra	− 0.068	0.621	0.913	1.700	0.828	0.040
Skeletal.group.Rib	− 0.093	0.582	0.873	2.151	0.796	0.007
Skeletal.group.Small bone	22.717	0.604	0.000	23.559	0.604	0.000
Skeletal.group.Stylopodials	0.683	0.948	0.471	1.856	1.117	0.097
Skeletal.group.Thoracic vertebra	0.994	0.564	0.078	2.723	0.772	0.000
Skeletal.group.Zeugopodials	0.669	1.223	0.584	1.340	1.390	0.335
Area.Pits.body	0.334	1.245	0.788	1.813	1.298	0.162
Area.Pits.dist shaft	− 0.796	0.631	0.207	0.099	0.644	0.878
Area.Pits.other	0.862	0.572	0.131	1.103	0.585	0.060
Area.Pits.spinous process	− 0.390	0.544	0.473	0.151	0.577	0.793
Area.Pits.transverse process	− 0.584	0.682	0.391	1.301	0.668	0.052
Area.Scores.dist shaft	4.444	2.108	0.035	4.184	2.153	0.052
Area.Scores.other	2.863	0.870	0.001	2.514	0.927	0.007
Area.Fracture.dist shaft	2.197	0.689	0.001	3.776	0.818	0.000
Area.Fracture.midshaft	0.495	0.959	0.606	3.973	0.972	0.000
Area.Fracture.other	0.782	0.638	0.220	1.235	0.794	0.120
Area.Fracture.prox shaft	0.116	0.996	0.907	3.933	0.996	0.000
Area.Fracture.spinous process	− 0.098	0.638	0.878	1.644	0.782	0.036
Area.Fracture.transverse process	− 1.083	0.647	0.094	1.387	0.850	0.103
Area.Crushing.dist shaft	2.749	0.663	0.000	0.876	0.903	0.332
Area.Crushing.other	1.273	0.545	0.019	0.752	0.738	0.308
Area.Crushing.transverse process	− 1.502	0.635	0.018	− 1.938	0.873	0.026
Area.Gral.Peeling.dist shaft	3.008	1.714	0.079	21.641	1.252	0.000
Area.Gral.Peeling.other	2.701	1.789	0.131	23.397	1.044	0.000
Area.Gral.Peeling.transverse process	1.567	1.772	0.376	20.906	1.088	0.000
Area.Bent.End.dist shaft	34.403	0.178	0.000	34.013	0.178	0.000
Area.Bent.End.other	18.248	0.374	0.000	19.066	0.381	0.000
Area.Fraying.dist shaft	− 5.244	0.157	0.000	− 5.926	0.157	0.000
Area.Fraying.other	25.665	0.183	0.000	26.315	0.183	0.000
Area.Cren.Edge.dist shaft	27.112	0.445	0.000	29.090	0.445	0.000
Area.Cren.Edge.other	0.872	1.632	0.593	2.874	1.641	0.080
Area.Cren.Edge.spinous process	0.137	1.437	0.924	3.109	1.462	0.033
Area.Cren.Edge.transverse process	− 0.062	1.669	0.970	2.960	1.662	0.075

By emphasizing the significance of *p*-values in identifying pertinent variables for classification purposes, we observe that within the quantitative variables, the number of pits (N. Pits) plays a crucial role in distinguishing between *Gyps fulvus* and *Vulpes vulpes* exerting notably a positive effect that favors the latter scavenger. However, the number of scores (N. Scores) has a highly favorable effect for griffon vulture dismissing both, *Ursus arctos* and *Vulpes vulpes*. There is a slight negative effect observed (*p*=0.068) when considering the impact of fractures on the classification of *Vulpes vulpes* but the same bone alteration does have a relatively significant effect on the classification of *Ursus ar*ctos. Conversely, the number of crushing exhibits a favorable effect on the classification of that large carnivore*.* The occurrence of longitudinal crushing or bent ends provides additional evidence supporting the classification of *Gyps fulvus* while an increase in the number of general peeling reduces the probability of *Vulpes vulpes* being classified as the scavenger. Finally, when considering the quantitative variables, the amount of furrowing provides supporting evidence for both *Ursus arctos* and *Vulpes vulpes*.

In terms of the qualitative variables, the presence of classic peeling provides some evidence favoring the classification of *Ursus arctos*. On the other hand, the occurrence of fraying decreases the probability of a bone being damaged by *Gyps fulvus*. When considering the skeletal group, the inclusion of cervical and lumbar vertebrae along with ribs contributes to an increased probability of *Vulpes vulpes* being identified as the scavenger. Moreover, thoracic vertebrae give strong evidence in favor of this last small carnivore. The same skeletal element provides supporting evidence for the classification of *Ursus arctos,* however, the cranial skeleton and girdles exhibit a relatively low probability, suggesting they are less indicative of this large carnivore as the main scavenger. Lastly, the presence of small bones strengthens the evidence against *Gyps fulvus* as the main taphonomic agent.

**Figure 5:**
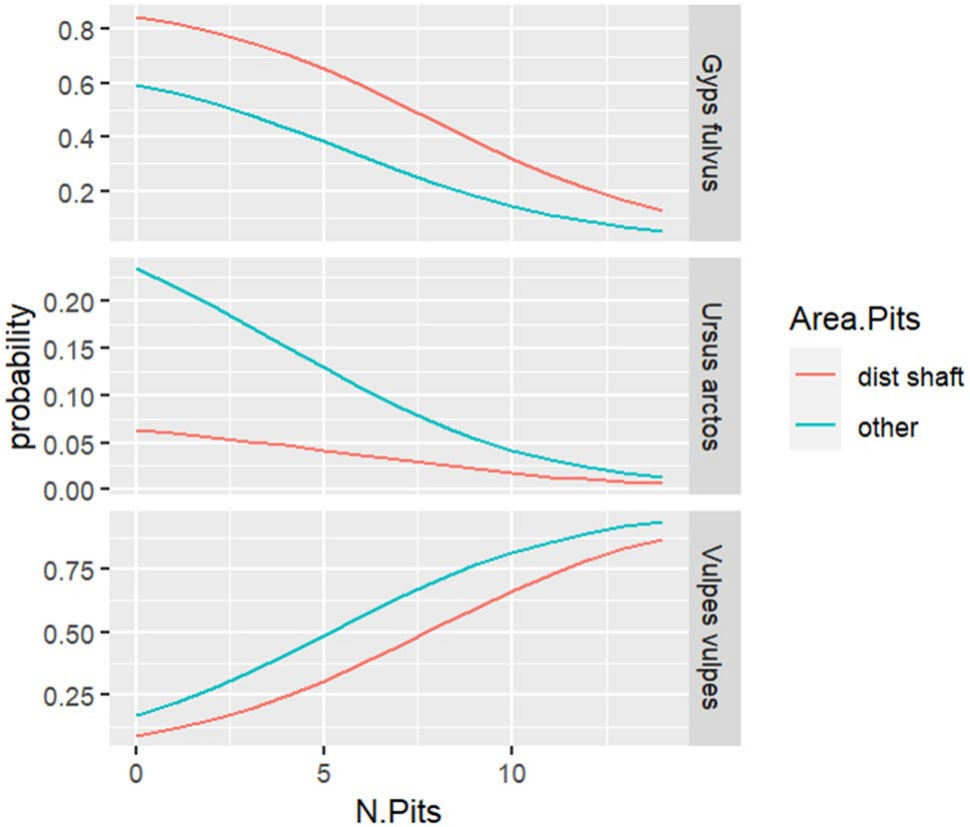
A graphical representation showing the likelihood distribution of pit quantities on ribs among the three distinct scavenger species.

**Figure 6:**
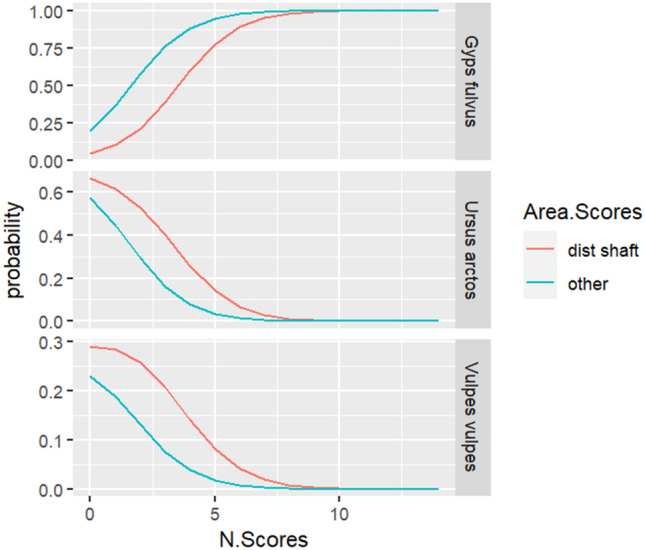
Plot illustrating the likelihood distribution of the number of scores on ribs among the three distinct scavenger species.

In relation to the dummy variables that are associated with the damaged areas of the bones, these simply collect changes in the starting point of the probability curve associated with the number of damages, that is, the case in which the bone is not damaged. For this reason, its interpretation is clearer when its effect is included together with the quantitative variable. Figures 5 and 6 show how the probability of classifying the three species of scavengers varies as the number of pits or scores increases in relation to the different bone damaged areas (dist shaft and other). We considered the bone being a rib and the age category being *inf* (infantile). Based on observations, in the absence of pits, *Gyps fulvus* is the most likely scavenger, followed by *Ursus arctos*, while the probability of *Vulpes vulpes* is very low. However, as the number of pits increases, the probability of *Vulpes vulpes* steadily rises and eventually approaches 1 for a large number of pits. In contrast, when examining the occurrence of scores, a small number of such types of modification are associated with the highest probability being attributed to *Ursus arctos*, followed by *Vulpes vulpes*. Nevertheless, as the number of scores increases, the probability of *Gyps fulvus* rapidly rises and approaches 1 when the number of scores exceeds 8.

## Discussion

Studies on obligate scavengers depict their taphonomic signature grounded primarily on shallow bone damage, mainly scores and striae, with the majority of bone surface modifications located on the diaphysis of long limb bones. Slight evidence such as irregularly shaped shallow scratches or linear surface striae without depth has also been documented principally on the crania and mandibles^26,27^ together with superficial single lines sometimes grouped and forming V or L-shapes^28^. However, the controversy emerges when describing a species-taphonomic hallmark based on a limited sample and, by extension, a limited data set. Our analysis results on 12 small-sized ungulate carcasses scavenged by griffon vultures point to a dissimilar scenario.

On the topic of bone induced-damage, ten different types of marks have been described and recorded in the present study without counting on subtypes and the well-founded division between pits and punctures as distinctiveness of vulture-made bone modification due to the help the latter split can provide when deciphering equifinality processes. As opposed to the low number of conspicuous pits and punctures that have been previously registered, our research shows an elevated frequency of these beak-marks ranging the third percentage-position only preceded by crushing and transversal fractures with a pit: puncture ratio of 17:39 (see Table 2). The location, shape and dimensions of isolated pits could mislead taphonomists on interpreting some specimens given that those marks located on the same anatomical areas are conventional in carnivore bone-altered assemblages. Therefore, the analysis of solely punctures and their combination with other type marks would help to individualize the responsible agency.

Some bone-unique made effects are listed here as v-grooves, u-grooves, longitudinal crushing and hollow out. Correspondingly, common grooves should be associated to v-grooves as a way to identify alleged scavenger especially on axial and girdles regions. Infantile individuals bear the highest percentage of those inflicted marks particularly on those regions together with the cranial skeleton of neonate specimens. The greater elasticity and less hardness of the compact bone tissue of these age groups compared to the pertinent in adult ones seem to facilitate this fact^67^. Additional singularities that would segregate small carnivores are, on the one hand, puncture, notches and groove-marks together with hollow out located on the costocondrale joint and recorded as individual damage. The precise forepaws and the elastic neck of the vulture family together with most of the vertebral column of the carcass articulated would be needed to access these particular points. Therefore, obvious physical reasons, for instance cranial shape and dentition, would prevent carnivores to inflict these peculiar marks neither on articulated thoracic cage nor on single individual ribs. On the other hand we registered pits in the medial area of the spinous process especially on thoracic vertebrae also retrieved from anatomical connected vertebral columns. The movements of the head together with the shape of the beak allow vultures to reach remote hidden small pieces of edible tissue and, unintentionally stamp their hallmark. Features such as this one and the fact that griffon vultures feed essentially on the softer viscera causes them to be called as gulpers^68^. Likewise, longitudinal crushing appears in remarkable percentages. A ratio of 4:7 (crushing: longitudinal crushing) is documented on the border of the ribs and a significant percentage is also documented on thoracic and lumbar vertebrae. We associate most longitudinal crushing with the eagerness of pecking soft tissue embedded between distal and mid-shafts of the ribs and vertebral transverse processes. From this perspective, the closeness towards the carcass seems to have an age-hierarchical character in the feeding bouts^69^ which leaves sub-adult, immature and juvenile griffon vultures competing for the last scraps and generating most of the previous inflicted bone-damage. The morphology of the complex head-neck system of these obligate scavengers performs in a variety of adaptations regarding its feeding strategy. Related to this, we were able to recover a stillborn calf (OB12) hollowed out from a skin hole in the lower abdomen with the solely result of a fractured hyoid-bone which illustrates the easiness of their long/elastic neck to reach remote and concealed scraps of soft tissue without hindrance. This particular morphologic feature would be associated in some cases with beak marks on both dorsal and ventral sides of skeletal elements such as ribs and transverse processes of vertebrae.

Nevertheless, the inference regarding the identification of the taphonomic agent turns out to be unambiguous when analyzing isolated incidences of cause-and-effect in some instances. As pointed out by Domínguez-Solera and Domínguez-Rodrigo^27^ the risk of potential equifinality problems increases as we widen the referential frameworks. In our case we identified some related-points such as pits/punctures together with scores on specimens of the axial skeleton and transversal fractures especially on the same skeletal region. Most of those alterations both, isolated or in association with different bone-type marks, have been recorded in small-sized ungulates scavenged by carnivores^6,7^.

In relation to the first point, as mentioned in the results section, we have observed that the distinguishing of pits and punctures located in certain skeletal regions can be beneficial when determining particular taphonomic agents. Moreover, and regarding pits/scores based on the quantitative variables, the multinomial model reveals that the number of pits plays a significant role in differentiating between griffon vulture and red fox, exerting a noticeable positive effect that favors the red fox as the main scavenger. On the other hand, the number of scores has a highly favorable effect for *Gyps fulvus*, while dismissing both *Ursus arctos* and *Vulpes vulpes*. Concerning the second aspect, we can observe a detrimental impact on the fox classification, with a higher probability of it with the brown bear and the vultures being the taphonomic agents as the number of fractures increases. There is currently insufficient evidence to differentiate between the last two scavengers, but expanding the dataset will facilitate obtaining more accurate outcomes.

## Conclusions

Previous studies show that griffon vultures reveal certain patterns primarily characterized by shallow damage to the bones, consisting mainly of scores and grooves located on the shafts of limb bones. Additionally, there is some evidence of irregularly shaped superficial scratches or linear grooves on the skulls and jaws, often forming V or L-shapes, along with superficial single lines that can occur individually or grouped together. The results we obtained reveal a range of bone alterations that are distinct from previous research. Most of the vulture-inflicted bone damage in our sample is concentrated in specific areas, such as the cranial skeleton, the girdles and the axial skeleton (bones with thin cortical).

Certain bone alterations observed in our research, such as crushing or transversal fractures, had not been previously documented, while others, like hollow out, v-grooves u-grooves and longitudinal crushing are being reported for the first time in this study as vulture-made bone-damage distinctiveness. These types of marks recorded in isolation or associated with specific type marks such as transversal fractures and/or pits/punctures/scores on small-sized ungulate carcasses point to obligate scavengers as the responsible taphonomic agent.

Like Domínguez-Solera and Domínguez-Rodrigo^27^, we also recognize a problem of equifinality concerning some bone damage such as pits and scores or transversal fractures. As starting point, the use of multinomial models has allowed distinguishing between the three carnivore species from the number of different taphonomic modifications and different bone damaged areas (distal shaft and other) using a bone (rib) of the axial skeleton of infantile individuals as an example. This model shows the probability of supporting evidence when classifying the three species of those scavengers from both quantitative and qualitative variables.

In conclusion, the findings and data from this research reveal a distinct pattern of griffon vulture scavenging small-sized ungulate carcasses. Nonetheless, it is imperative to conduct additional neo-taphonomic research that broadens the sample, encompassing not only a larger dataset but also diverse age categories in relation to ungulate carcasses and the interaction of different obligate and facultative avian scavengers. It is also essential to combine the results from the taphonomic studies with statistical analyses when approaching equifinality problems as this combination is what can assist us in individualizing the griffon vulture taphonomic signature in archaeological faunal assemblages.

## Data Availability

All data used in this study have been included in the article. Additional data related to this paper may be requested from the authors on request. Correspondence and requests for materials should be addressed to R.B. and M.A.

## References

[1] Pickering TR, Wallis J (1997). Bone modifications resulting from captive chimpanzee mastication: Implications for the interpretation of Pliocene archaeological faunas. J. Archaeol. Sci..

[2] Domínguez-Rodrigo M, Piqueras A (2003). The use of tooth pits to identify carnivore taxa in tooth-marked archaeofaunas and their relevance to reconstruct hominid carcass processing behaviours. J. Archeol. Sci..

[3] Pobiner BL, De Silva J, Sanders WJ, Mitani JC (2007). Taphonomic analysis of skeletal remains from chimpanzee hunts at Ngogo, Kibale National Park, Uganda. J. Hum. Evol..

[4] Domínguez-Rodrigo M, Gidna A, Yravedra J, Mushiba C (2012). A comparative neo-taphonomic study of felids, hyaenids and canids: An analogical framework based on long bone modification patterns. J. Taphon..

[5] Arilla M, Rosell J, Blasco R, Domínguez-Rodrigo M, Pickering TR (2014). The “bear” essentials: actualistic research on *Ursus arctos arctos* in the Spanish Pyrenees and its implications for paleontology and archaeology. PLoS One.

[6] Arilla M, Rosell J, Blasco R (2018). Contributing to characterize wild predator behavior: Consumption pattern, spatial distribution and bone damage on ungulate carcasses consumed by red fox (*Vulpes vulpes*). Archaeol. Anthrop. Sci..

[7] Arilla M, Rufà A, Rosell J, Blasco R (2019). Small carnivores’ cave-dwelling: Neo-taphonomic study of a badger (*Meles meles*) sett and its archaeological implications. Hist. Biol..

[8] Arilla M, Rosell J, Blasco R (2020). A neo-taphonomic approach to human campsites modified by carnivores. Sci. Rep..

[9] Rosell J, Blasco R, Arilla M, Fernández-Jalvo Y (2019). Very human bears: Wild brown bear neo-taphonomic signature and its equifinality problems in archaeological contexts. Quat. Int..

[10] Blasco R, Arilla M, Domínguez-Rodrigo M, Andrés M, Ramírez-Pedraza I (2020). Who peeled the bones? An actualistic and taphonomic study of axial elements from the Toll Cave Level 4, Barcelona Spain. Quat. Sci. Rev..

[11] Kerbis-Peterhans, J. C. *The Roles of Porcupines, Leopards and Hyaenas in Ungulate Carcass Dispersal: IMPLICATIONS for Paleoanthropology* (PhD. dissertation. University of Chicago, Chicago, 1990).

[12] Cavallo, J. A. *Tree-Cached Leopard Kills and Early Hominid Foraging Strategies: An Actualistic Study* (PhD dissertation, Rutgers University, New Brunswick, 1997).

[13] Skinner JD, Haupt MA, Hoffmann M, Dott HM (1998). Bone collecting by brown hyaenas *Hyaena brunnea* in the Namib Desert: Rate of accumulations. J. Archaeol. Sci..

[14] Domínguez-Rodrigo M (1999). Flesh availability and bone modification in carcasses consumed by lions. Palaeogeogr. Palaeogeogr. Palaeogeogr..

[15] Pickering TR, Domínguez-Rodrigo M, Egeland CP, Brain CK (2004). Beyond leopards: Tooth marks and the contribution of multiple carnivore taxa to the accumulation of the Swartkrans member 3 fossil assemblage. J. Hum. Evol..

[16] Faith JT, Marean CW, Behrensmeyer AK (2007). Carnivore competition, bone destruction, and bone density. J. Archaeol. Sci..

[17] Prendergast ME, Domínguez-Rodrigo M (2008). Taphonomic analyses of a hyena den and a natural-death assemblage near Lake Eyasi (Tanzania). J. Taphon..

[18] Sala N, Arsuaga JL (2018). Regarding beasts and humans: A review of taphonomic works with living carnivores. Quat. Int..

[19] Bochenski ZM, Tomek T, Tornberg R, Wertz K (2009). Distinguishing nonhuman predation on birds: Pattern of damage done by the white-tailed eagle *Haliaetus albicilla*, with comments on the punctures made by the golden eagle *Aquila chrysaetos*. J. Archeol. Sci..

[20] Armstrong A, Avery G (2014). Taphonomy of Verreaux's Eagle (*Aquila verreauxii*) prey accumulations from the Cape Floral Region, South Africa: Implications for archaeological interpretations. J. Archeol. Sci..

[21] Marín-Arroyo AB, Margalida A (2012). Distinguishing bearded vulture activities within archaeological contexts: Identification guidelines. Int. J. Osteoarchaeol..

[22] Lloveras L, Thomas R, Lourenço R, Caro J, Diaset A (2014). Understanding the taphonomic signature of Bonelli’s Eagle (*Aquila fasciata*). J. Archeol. Sci..

[23] Lloveras L, Cosso A, Solé J, Claramunt-López B, Nadal J (2017). Taphonomic signature of golden eagles (*Aquila chrysaetos*) on bone prey remains. Hist. Biol..

[24] Alonso G, Rufà A, Arilla M, Blasco R (2019). Taphonomic signature of the Eurasian eagle-owl (Bubo bubo) on the avian accumulation of Cau del Duc (Lleida, Spain). Hist. Biol..

[25] Cáceres, I. *Tafonomía de yacimientos antrópicos en Karst. Complejo Galería (Sierra de Atapuerca, Burgos), Vanguard Cave (Gibraltar) y Abric Romaní (Capellades, Barcelona)* (PhD dissertation, Universitat Rovira i Virgili, 2002).

[26] Reeves NM (2009). Taphonomic effects of vulture scavenging. J. Forensic Sci..

[27] Domínguez-Solera S, Domínguez-Rodrigo M (2011). A taphonomic study of a carcass consumed by griffon vultures (*Gyps ful*vus) and its relevance for the interpretation of bone surface modifications. Archaeol. Anthropol. Sci..

[28] Fetner RA, Sołtysiak A (2013). Shape and distribution of griffon vulture (*Gyps fulvus*) scavenging marks on a bovine skull. J. Taphon..

[29] Tyrberg, T. *Pleistocene Birds of the PALEARCTIC: A Catalogue* (Publications of the Nuttall Ornitho-logical Club, no. 27, Cambridge, Massachusetts, 1998).

[30] Robert I, Vigne J (2002). The bearded vulture (*Gypaetus barbatus*) as an accumulator of archaeological bones. Late glacial assemblages and present-day reference data in Corsica (Western Mediterranean). J. Archaeol. Sci..

[31] Marín-Arroyo A, Fosse P, Vigne JD (2009). Probable evidences of bone accumulation by Pleistocene bearded vulture at the archaeological site of El Mirón Cave (Spain). J. Archeol. Sci..

[32] BirdLife International. *State of the World’s Birds: Taking the Pulse of the Planet* (Cambridge, UK, 2018).

[33] McClure CJ, Westrip JR, Johnson JA, Schulwitz SE, Virani MZ (2018). State of the world’s raptors: Distributions, threats, and conservation recommendations. Biol. Conserv..

[34] Bennett PM, Owens IPF (1997). Variation in extinction risk among birds: Chance or evolutionary predisposition?. Proc. Biol. Sci..

[35] Şekercioğlu ÇH, Daily GC, Ehrlich PR (2004). Ecosystem consequences of bird declines. PNAS.

[36] Safford R, Andevski J, Botha A, Bowden CGR, Crockford N (2019). Vulture conservation: The case for urgent action. Bird Conserv. Int..

[37] Moreno-Opo R, Trujillano A, Margalida A (2016). Behavioural coexistence and feeding efficiency drive niche partitioning at carcasses within the guild of European avian scavengers. Behav. Ecol..

[38] Morales-Reyes Z, Pérez-García JM, Moleón M, Botella F, Carrete M (2017). Evaluation of the network of protection areas for the feeding of scavengers (PAFs) in Spain: From biodiversity conservation to greenhouse gas emission savings. J. Appl. Ecol..

[39] Alarcón PAE, Lambertucci SA (2018). A three-decade review of telemetry studies on vultures and condors. Mov. Ecol..

[40] Aguilera-Alcalá N, Morales-Reyes Z, Martín-López B, Moleón M, Sánchez-Zapata JA (2020). Role of scavengers in providing non-material contributions to people. Ecol. Indic..

[41] García-Jiménez R, Morales-Reyes Z, Pérez-García JM, Margalida A (2021). Economic valuation of non-material contributions to people provided by avian scavengers: Harmonizing conservation and wildlife-based tourism. Ecol. Econ..

[42] Lambertucci S, Margalida A, Amar A, Ballejo F, Blanco G (2021). Presumed killers? Vultures, stakeholders, misperceptions and fake news. Conserv. Sci. Pract..

[43] Oliva-Vidal P, Hernández-Matías A, García D, Colomer MÀ, Real J (2022). Griffon vultures, livestock and farmers: Unraveling a complex socio-economic ecological conflict from a conservation perspective. Biol. Conserv..

[44] Arrondo E, Guido J, Oliva-Vidal P, Margalida A, Lambertucci S (2023). From pyrenees to andes: The relationship between of transhumant livestock and vultures. Biol. Conserv..

[45] Morant J, Arrondo E, Sánchez-Zapata JA, Donázar JA, Cortés-Avizanda A (2023). Large-scale movement patterns in a social vulture are influenced by seasonality, sex, and breeding región. Ecol Evol..

[46] Haglund, W. & Sorg, M. H. *Forensic Taphonomy. The Postmortem Fate of Human Remains* (CRC Press, 1996)

[47] Houck MM (2017). Forensic Anthropology.

[48] Spradley MK, Hamilton MD, Giordano A (2012). Spatial patterning of vulture scavenged human remains. Forensic Sci. Int..

[49] Dabbs GR, Martin DC (2013). Geographic variation in the taphonomic effect of vulture scavenging: The case for Southern Illinois. J. Forensic Sci..

[50] Lewis KN (2018). The Effects of Clothing on Vulture Scavenging and Spatial Distribution of Human Remains in Central Texas.

[51] Ninot JM, Carrillo E, Font X, Carreras J, Ferré A (2007). Altitude zonation in the Pyrenees. A geobotanic interpretation. Phytocoenologia.

[52] Colomer MA, Margalida A, Sanuy D, Pérez-Jiménez MJ (2011). A bio-inspired computing model as a new tool for modelling ecosystems: The avian scavengers as a case study. Ecol. Model..

[53] Oliva-Vidal, P., Sebastián-González, E. & Margalida, A. Scavenging in changing environments: Woody encroachment shapes rural scavenger assemblages in Europe. *Oikos*. e09310 (2022b).

[54] Del Hoyo, J., Elliott, A. & Sargatal, J. *Handbook of the Birds of the World* (Lynx Edicions, Vol. 2., Barcelona, 1994).

[55] Xirouchakis SM, Mylonas M (2007). Breeding behaviour and parental care in the Griffon Vulture *Gyps fulvus* on the island of Crete (Greece). Ethol. Ecol. Evol..

[56] Margalida A, Bertran J (2000). Breeding behaviour of the Bearded Vulture (*Gypaetus barbatus*): Minimal sexual differences in parental activities. Ibis.

[57] Delgado-González A, Cortés-Avizanda A, Serrano D, Arrondo E, Duriez O (2022). Apex scavengers from different European populations converge at threatened savannah landscapes. Sci. Rep..

[58] Maguire JM, Pemberton D, Collett MH (1980). The Makapansgat limeworks grey breccia: Hominids, hyaenas, hystricids or hillwash. Palaeontol. Afr..

[59] Haynes G (1980). Evidence of carnivore gnawing on Pleistocene and recent mammalian bones. Paleobiol..

[60] Blumenschine RJ, Selvaggio M (1988). Percussion marks on bone surfaces as a new diagnostic of hominid behavior. Nature.

[61] Andrews P (1995). Experiments in Taphonomy. J. Archeol. Sci..

[62] White, T.D. *Prehistoric Cannibalism at Mancos 5MTURM-2346*. (Princeton University Press, 1992).

[63] Pickering TR, Domínguez-Rodrigo M, Heaton JL, Yravedra J, Barba R (2013). Taphonomy of ungulate ribs and the consumption of meat and bone by 1.2-million-year-old hominins at Olduvai Gorge Tanzania. J. Archeol. Sci..

[64] Efron, B. & Hastie, T. *Computer Age Statistical Inference* (Cambridge University Press, 2016).

[65] Venables WN, Ripley BD (2002). Modern Applied Statistics with S.

[66] Wickham H (2016). ggplot2: Elegant Graphics for Data Analysis.

[67] Nafei A, Danielsen CC, Linde F, Hvid I (2000). Properties of growing trabecular ovine bone. Part 1: mechanical and physical properties. J. Bone Jt. Surg..

[68] Böhmer C, Prevoteau J, Duriez O, Abourachid A (2020). Gulper, ripper and scrapper: Anatomy of the neck in three species of vultures. J. Anat..

[69] Moreno-Opo R, Trujillano A, Margalida A (2020). Larger size and older age confer competitive advantage: Dominance hierarchy within European vulture guild. Sci. Rep..

